# The Role of Body Mass Index in Outcomes of Radial Shock Wave Therapy for Adhesive Capsulitis

**DOI:** 10.3390/biomedicines13092117

**Published:** 2025-08-29

**Authors:** Diana-Lidia Tache-Codreanu, Iuliana David, Mihai-Andrei Butum-Cristea, Ana-Maria Tache-Codreanu, Claudia-Camelia Burcea, Elena Rusu, Andrei Tache-Codreanu, Rodica Olteanu, Teodor Dan Poteca, Corina Sporea

**Affiliations:** 1 Medical Rehabilitation Department, Colentina Clinical Hospital, Stefan cel Mare Street No. 19–21, 020125 Bucharest, Romania; julexim@gmail.com (I.D.); mihai5020@gmail.com (M.-A.B.-C.); rodicaolteanu@hotmail.com (R.O.); teo_poteca@yahoo.com (T.D.P.); 2 Faculty of Medicine and Farmacy, University of Medicine and Pharmacy “Carol Davila”, 37 Dionisie Lupu Street, 020021 Bucharest, Romania; ana-maria.tache2024@stud.umfcd.ro (A.-M.T.-C.); corina.sporea@gmail.com (C.S.); 3 Department of Preclinical Disciplines, Faculty of Medicine, Titu Maiorescu University, 031593 Bucharest, Romania; elenarusu98@yahoo.com; 4 Doctoral School, The National University of Theatre and Film “I.L. Caragiale”, 75–77 Matei Voievod Street, 021452 Bucharest, Romania; andrei.tachecodreanu@unatc.ro; 5 National University Center for Children Neurorehabilitation “Dr. Nicolae Robanescu”, 44 Dumitru Minca Street, 041408 Bucharest, Romania

**Keywords:** radial shockwave therapy, adhesive capsulitis, body mass index, treatment outcomes, correlation

## Abstract

**Background**: Radial shock wave therapy (RSWT) has increasingly been integrated into treatment protocols for adhesive capsulitis. While associations with diabetes and other systemic disorders are well documented, the role of obesity remains underexplored, particularly in relation to RSWT outcomes. **Methods**: Forty patients with adhesive capsulitis completed a 10-day treatment protocol combining RSWT with conventional physiotherapy. Pain (VAS), disability (SPADI), and range of motion (ROM) were assessed at baseline and immediately after treatment. At one-month follow-up, VAS and SPADI were reassessed alongside the Patient Global Impression of Change (PGIC). Correlations between body mass index (BMI) and clinical outcomes were analyzed, and potential confounding effects of comorbidities and affected-side dominance were examined. Clinical relevance was assessed using minimal clinically important differences (MCID) and effect sizes (Cohen’s d). **Results**: All clinical outcomes improved significantly post-treatment and at follow-up, with most changes exceeding MCID thresholds and showing large effect sizes. Higher BMI was significantly correlated with greater improvements in SPADI, VAS, shoulder extension, and internal rotation. Most comorbidities were negatively associated with outcomes, except neurologic conditions, which supported mobility improvement. **Conclusions**: RSWT appears effective in alleviating symptoms of adhesive capsulitis. The observed association between higher BMI and greater mobility improvement suggests potential benefits in overweight and obese patients. These findings warrant further investigation.

## 1. Introduction

Adhesive capsulitis—also referred to as frozen shoulder or arthrofibrosis—is a painful and debilitating condition resulting from excessive scar tissue formation and adhesions around the glenohumeral joint [1,2]. It affects approximately 2% to 5% of the general population and leads to progressive stiffness, pain, restriction of both active and passive shoulder motion, and overall joint dysfunction [2,3].

There is a well-established correlation between upper limb functionality and independence in both activities of daily living (ADLs) and instrumental activities of daily living (IADLs), underscoring the broader impact of this condition on quality of life and autonomy [4,5,6].

The typical clinical course of adhesive capsulitis is classically divided into three phases. The initial painful phase, which typically lasts between 2 and 9 months, is characterized by progressive shoulder pain, often more pronounced at night. During the subsequent freezing phase—lasting approximately 4 to 12 months—pain may gradually subside; however, this phase is marked by a significant loss of glenohumeral joint mobility. Finally, the thawing phase, which may span 5 to 26 months, is associated with a gradual recovery of range of motion and resolution of symptoms. If symptoms persist beyond this final phase, they are generally mild, with pain remaining the most limiting factor [1,7]. Although spontaneous resolution is possible, recovery is often incomplete without targeted intervention.

The treatment of adhesive capsulitis primarily focuses on pain management, with physiotherapy remaining the cornerstone of conservative care. Traditional modalities such as thermal agents, electrotherapy, therapeutic exercise, and manual therapy have been widely used in musculoskeletal rehabilitation. In recent years, these have increasingly been complemented by non-invasive physical agents, including radial extracorporeal shock wave therapy (RSWT), high-intensity laser therapy (HIL), and capacitive–resistive electric transfer therapy (TECAR), all of which have shown promising results. RSWT is believed to promote revascularization, reduce inflammation, and stimulate connective tissue healing, thereby improving shoulder mobility and reducing disability [1,8,9]. HIL, through deep-tissue photothermal effects, may enhance analgesia and accelerate recovery by stimulating cellular metabolism and microcirculation [10]. TECAR therapy, which modulates pain and improves soft tissue extensibility via endogenous heat production, has also been associated with favorable outcomes in shoulder disorders [11,12,13,14,15]. The effectiveness—and in some studies, the superiority—of RSWT compared to conventional conservative treatments has been demonstrated in several clinical trials [9,16,17,18,19,20,21,22,23]. In cases where non-invasive management fails, patients are typically advised to consider corticosteroid injections or surgical interventions such as capsular release [1].

Around 70% of affected individuals are women. Although the role of sex in the etiology and development of adhesive capsulitis remains unclear, this disparity may be associated with hormonal influences or immune system differences [1,2,3]. The mean age of onset is approximately 55 years, which coincides with a stage of life when the general population shows an increased incidence of systemic conditions such as diabetes mellitus and thyroid disorders [22]. These, along with cerebrovascular disease, coronary artery disease, autoimmune diseases, and Dupuytren’s disease, have been proposed as potential risk factors for adhesive capsulitis. Other proposed associations include previous trauma, HLA-B27 positivity, dyslipidemia, and prolonged immobilization of the glenohumeral joint [2]. Notably, the non-dominant limb is more frequently affected, which may relate to relative immobilization resulting from compensatory behaviors that typically favor increased use of the dominant side [24].

Frozen shoulder has been increasingly recognized as a condition closely linked to metabolic dysfunction, particularly a state of metabolic inflammation characterized by low-grade chronic systemic inflammation driven by adipocytokines and insulin resistance [25,26,27]. Obesity contributes to this pro-inflammatory milieu by promoting the release of cytokines such as interleukin-6 (IL-6), tumor necrosis factor-alpha (TNF-α), and C-reactive protein (CRP), which may adversely affect both the synovial environment and the fibrotic response within the joint capsule [28,29]. Recent evidence further suggests the existence of a distinct metabolic phenotype of adhesive capsulitis, in which obesity and metabolic syndrome play a central pathophysiological role, particularly through systemic dysmetabolism and inflammation, even in younger patients [30,31]. Despite these links, obesity remains underrepresented in clinical studies on adhesive capsulitis. Recent large-scale cohort data from Korea suggest that obesity independently increases the risk of adhesive capsulitis in younger adults (20–40 years), whereas in older individuals, the risk appears to be significantly elevated only in the presence of metabolic comorbidities such as diabetes and hyperlipidemia [32]. Regarding treatment outcomes, a study by Barbosa et al. reported that diabetes was associated with poorer response to conservative care and increased likelihood of surgical intervention. However, body mass index (BMI) alone did not significantly influence treatment efficacy [33]. Moreover, a meta-analysis by Allgussin et al. indicated that obesity may adversely affect postoperative shoulder outcomes, including range of motion and pain [34]. Excess body weight may also contribute to reduced physical endurance and prolonged recovery time during both the acute inflammatory phase and the chronic fibrotic phase of adhesive capsulitis, further complicating rehabilitation [35]. To date, the influence of obesity on the response to RSWT in adhesive capsulitis remains unexplored.

From a physical therapy perspective, higher BMI may attenuate the penetration of mechanical energy delivered via RSWT due to greater subcutaneous tissue thickness [36]. Additionally, excess body weight may also contribute to altered pain threshold and reduced exercise tolerance, yielding prolonged recovery time during both the acute inflammatory phase and the chronic fibrotic phase of adhesive capsulitis, further complicating rehabilitation and overall treatment efficacy [35,37]. To date, the influence of obesity on the response to RSWT in adhesive capsulitis remains unexplored.

The aim of this study was to evaluate the therapeutic effect of RSWT in patients with adhesive capsulitis and to examine whether BMI influences baseline symptom severity or clinical response to treatment.

## 2. Materials and Methods

### 2.1. Study Design and Ethical Considerations

This retrospective observational study was conducted at Coletina Clinical Hospital in Bucharest between December 2024 and May 2025 and included 40 patients clinically diagnosed with adhesive capsulitis. All participants were informed of potential risks and benefits and provided written informed consent in accordance with institutional guidelines. The study was approved by the Ethics Committee of Colentina Clinical Hospital (approval number 16/27 July 2017), in accordance with the Declaration of Helsinki.

### 2.2. Participants

Inclusion criteria were as follows: age between 18 and 80 years; a clinical diagnosis of adhesive capsulitis characterized by recurrent shoulder pain and functional limitation; and an inadequate response to at least four weeks of pharmacological treatment, including oral or local nonsteroidal anti-inflammatory drugs (NSAIDs) or muscle relaxants.

All patients were in the tardive subacute phase of adhesive capsulitis, with symptom duration between 3 and 6 months—a stage typically associated with both pain and restricted mobility. Prior to enrollment, patients had received pharmacological treatment only; no physical therapy, injections, or other interventions were administered. Specifically, no corticosteroid injection into the affected shoulder was permitted within the previous six months, and no other intra-articular or periarticular injections had been performed. To enable subgroup analysis, only patients with a BMI ≥ 18.5 were included. Efforts were made to ensure at least 10 participants in each BMI category: normal weight (18.5–24.9), overweight (25.0–29.9), and obese (≥30.0) [32,38,39]. Exclusion criteria were the following: clinical or imaging evidence of rotator cuff tear; age <18 or >80 years; prior surgery on the affected shoulder; pregnancy; presence of pacemakers; coagulation disorders or ongoing anticoagulant therapy; neoplastic or systemic inflammatory diseases; osteomyelitis; acute bursitis; rheumatoid arthritis or other connective tissue disorders; corticosteroid injection into the affected shoulder within the previous six months; and non-adherence to the prescribed physiotherapy and kinesiotherapy protocol [40,41,42].

### 2.3. Interventions

All patients received a standardized conservative treatment program consisting of ten consecutive daily sessions of conventional physiotherapy, which included transcutaneous electrical nerve stimulation, laser therapy, and magnetotherapy for analgesic and anti-inflammatory purposes [10]. Patients were advised to follow home-based physical therapy guidelines, including relative rest of the affected shoulder and pendulum exercises to prevent capsular contracture. Additionally, each patient underwent five weekly sessions of RSWT, administered using the Magnum applicator (BTL Industries Ltd., Prague, Czech Republic).

RSWT sessions began at an energy level of 2.5–3.0 bar and were gradually increased to a maximum of 6.0 bar over the first three sessions, depending on patient tolerance. The frequency ranged from 10 to 15 Hz, with 2000 pulses delivered per session. Five weekly sessions were administered. Prior to each application, coupling gel was used to optimize energy transmission. Hyperalgesic areas were avoided to minimize discomfort. Treatments were applied with the patient seated, shoulder abducted to 45°, elbow flexed to 90°, and forearm supported on a flat surface.

### 2.4. Outcome Measures

Clinical outcomes were assessed using four validated instruments: the Shoulder Pain and Disability Index (SPADI), the Visual Analog Scale (VAS) for pain, standardized goniometric measurement of shoulder range of motion (ROM), and the Patient Global Impression of Change (PGIC) scale [42,43,44,45,46].

SPADI, VAS, and ROM were evaluated at baseline and immediately after treatment, while SPADI and VAS were reassessed at the one-month follow-up [47]. PGIC was assessed only at the one-month follow-up. The primary outcome measures were SPADI and VAS, selected for their relevance in capturing both pain intensity and functional limitation, which are central to adhesive capsulitis.

#### 2.4.1. Shoulder Pain and Disability Index (SPADI)

The SPADI was chosen as a primary outcome due to its established validity, reliability, and responsiveness in patients with shoulder pathology. It comprises two subscales: pain (five items) and disability (eight items), each rated on a 0–10 numerical scale. The total score is expressed as a percentage, with higher values indicating greater impairment. SPADI has demonstrated excellent internal consistency (Cronbach’s α > 0.90) and test-retest reliability across a range of shoulder conditions, including adhesive capsulitis. Its responsiveness to clinical change makes it particularly appropriate for short-term intervention studies such as the present one.

#### 2.4.2. Visual Analog Scale (VAS)

The VAS was used alongside SPADI to assess pain intensity. This unidimensional tool consists of a 10 cm horizontal line anchored by “no pain” (0 cm) and “worst imaginable pain” (10 cm). Patients marked the point that best represented their current pain at rest. The score was determined by measuring the distance in centimeters from the “no pain” anchor. VAS is widely accepted for its simplicity, sensitivity to change, and relevance in musculoskeletal pain research. It complements functional assessments like SPADI by capturing the subjective experience of pain.

The combined use of SPADI and VAS provides a comprehensive evaluation of both functional limitation and pain severity, enabling a nuanced assessment of RSWT’s therapeutic effects in adhesive capsulitis. These instruments were applied at baseline, immediately post-intervention, and—except for ROM—at one-month follow-up.

#### 2.4.3. Patient Global Impression of Change (PGIC)

The PGIC was included as a complementary measure to capture patients’ subjective perception of overall improvement. PGIC is a validated, single-item scale ranging from 1 (“no change or condition has worsened”) to 7 (“a great deal better and a considerable improvement that has made all the difference”). It reflects the patient’s perspective on change in symptoms, function, and quality of life since the start of treatment.

In adhesive capsulitis, where pain and disability significantly impact daily life, PGIC offers valuable insight into the perceived effectiveness of treatment beyond clinical metrics. Unlike condition-specific instruments such as SPADI or VAS, PGIC integrates physical, emotional, and contextual domains, aligning with patient-centered care models. In this study, PGIC was administered at the one-month follow-up to allow adequate time for treatment effects to consolidate. Despite its subjectivity, PGIC has shown strong associations with functional recovery and patient satisfaction in musculoskeletal rehabilitation, supporting its use as a global outcome indicator.

#### 2.4.4. Range of Motion (ROM)

ROM was assessed to objectively evaluate joint mobility and functional restoration. Assessment included five standard directions: flexion, abduction, extension, internal rotation, and external rotation. These movements were chosen based on their consistent impairment in adhesive capsulitis and their clinical relevance. Measurements were performed by a single trained physiotherapist using a standardized universal goniometer and a reproducible positioning protocol to ensure inter-session consistency.

Patients were assessed in a seated position with neutral trunk alignment and scapular stabilization. Flexion and abduction were measured in the sagittal and frontal planes, respectively. Extension, internal rotation, and external rotation were assessed with the arm abducted to 90° and the elbow flexed at 90°, following established orthopedic examination guidelines. All movements were performed actively, with the best of three consistent trials recorded. ROM values were expressed in degrees.

ROM was assessed at baseline and immediately after the intervention. Due to logistical constraints, it was not reassessed at follow-up. However, immediate post-treatment improvements in ROM were considered indicative of short-term functional gains. In the context of adhesive capsulitis—where capsular contracture and muscle guarding restrict motion—goniometric ROM assessment remains a critical component of functional evaluation. Together with SPADI, VAS, and PGIC, ROM measurements provide a multidimensional view of clinical outcomes following RSWT.

### 2.5. Clinical Relevance of Outcomes

To assess the clinical relevance of the observed changes, minimal clinically important differences (MCID) were considered for each outcome measure. According to previous studies, the MCID is estimated to be 8–13 points for the SPADI questionnaire [48], 1.5–2.0 points on a 10-point scale for the Visual Analogue Scale (VAS) [49], and approximately 10–20° for shoulder range of motion, depending on the specific movement assessed [50,51]. In addition to MCID thresholds, the magnitude of change was further evaluated using standardized effect sizes to support the practical relevance of the findings.

### 2.6. Baseline and Moderating Variables

BMI was recorded at baseline and categorized according to World Health Organization guidelines: normal weight (18.5–24.9), overweight (25.0–29.9), and obese (≥30.0). Analyses explored the relationship between BMI and baseline symptom severity (SPADI, VAS, ROM), as well as its potential moderating effect on clinical improvement following treatment. In addition to BMI, the influence of comorbidities and affected-side dominance (dominant vs. non-dominant limb) on treatment outcomes was assessed. Comorbidities were extracted from patient records and categorized into clinical domains based on the ICD-10 criteria and clinical relevance. The following categories were defined:

- Musculoskeletal diseases (e.g., cervical/lumbar discopathies, tendinopathies, gonarthrosis, osteoporosis, carpal tunnel syndrome, meniscal and knee ligament injuries, scoliosis, epicondylitis, vertebral fractures, finger amputation, joint arthroplasties, acromioclavicular arthrosis, coxarthrosis, polyarthrosis of the hand);
- Cardiovascular diseases (e.g., arterial hypertension, atrioventricular block, angina pectoris, varicose veins);
- Digestive diseases (e.g., hiatal or inguinal hernia, hepatic steatosis);
- Endocrinologic diseases (e.g., thyroiditis);
- Infectious diseases (e.g., chronic hepatitis B);
- Metabolic diseases (e.g., dyslipidemia, diabetes mellitus, vitamin B1, D3, or folic acid deficiency);
- Neurologic diseases (neuropsychiatric) (e.g., polyneuropathies, vertigo, demyelinating lesions, anxiety, depressive syndrome, radiculopathies, syringomyelia);
- Renal diseases (e.g., chronic kidney disease, nephrolithiasis);
- Hematologic diseases (e.g., chronic anemia);
- Pulmonary diseases (e.g., asthma).

Comorbidities were analyzed as binary variables (presence/absence). A separate variable was created to identify musculoskeletal conditions affecting the same upper limb as the treated shoulder.

Additionally, potential associations between BMI categories and comorbidity domains were explored to identify confounding relationships.

### 2.7. Sample Size Calculation

A minimum sample size of 8 participants was calculated to detect significant Pre–Post changes in VAS scores. The following formula was used to achieve 80% statistical power [52]:

$$Sample\;size=\frac{2{SD}^{2}\cdot {\left(1.96+0.84\right)}^{2}}{d^{2}},$$

where effect size *d* = 1.5, standard deviation *SD* = 1.02 (based on Kim et al.) [53].

To enable subgroup analysis by BMI, at least 10 patients per category were targeted, resulting in a final sample of 40 participants for this pilot investigation.

### 2.8. Statistical Analysis

Data were analyzed using a custom MATLAB script (MATLAB R2021a, The MathWorks, Inc., Natick, MA, USA). Normality was assessed using the Shapiro–Wilk test, and non-parametric tests were applied due to non-normal distributions (*p* < 0.05 for all variables). Descriptive statistics were reported as medians and interquartile ranges (IQR). Within-subject comparisons across time points were evaluated using the Friedman test, with post-hoc Wilcoxon signed-rank tests and Bonferroni corrections. Additionally, Cohen’s d was calculated to estimate the effect size of within-subject changes between pre- and post-treatment values. The pooled standard deviation of baseline and post-treatment scores was used in the denominator.

Associations between BMI and outcome variables (both baseline and change scores) were analyzed using Spearman’s rank correlation and the Kruskal–Wallis test. The Mann–Whitney U test was used to assess the impact of comorbidities and limb dominance on clinical outcomes, as well as to compare BMI distributions between patients with and without specific comorbidities.

For variables where initial correlation or group comparison suggested a potential association with BMI (typically *p* < 0.10), multiple linear regression analyses were conducted using BMI category as a categorical predictor (reference: normal weight). In cases where the overall model showed a significant or near-significant effect (typically *p* < 0.10), post-hoc analyses were performed to interpret individual group differences. Coefficients representing overweight and obese patients were compared against the normal weight reference group to determine which subgroup primarily drove the observed effect. Statistical significance was set at *p* < 0.05 (two-tailed). 

## 3. Results

A total of 40 patients with adhesive capsulitis were included in the study, comprising 25 women and 15 men. The median age of participants was 68.5 years (IQR: 54.3–73.0), and the median BMI was 26.13 kg/m^2^ (IQR: 23.24–30.86). Based on WHO criteria, 14 patients were classified as having normal weight, 12 as overweight, and 12 as obese.

All participants completed the treatment protocol and follow-up assessments. No dropouts or therapy-related adverse events were reported, and the intervention was well tolerated across all BMI categories.

Significant improvements were observed in SPADI and VAS scores immediately post-treatment, with additional gains noted at one-month follow-up. These improvements were confirmed by both the Wilcoxon signed-rank test and the Friedman test, indicating consistent and robust effects over time (Table 1).

**Figure 1:**
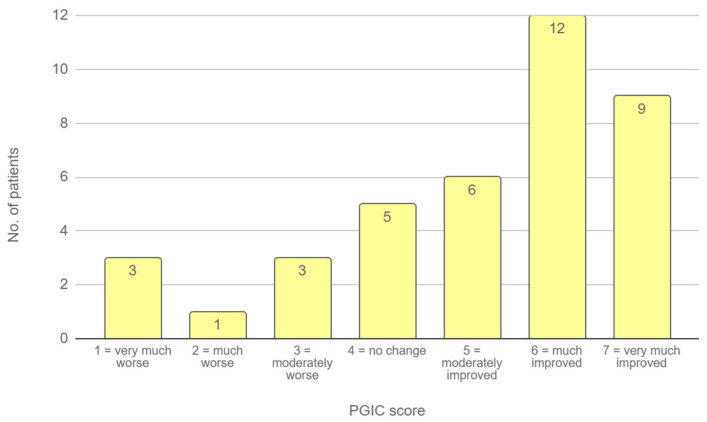
PGIC scores at follow-up. The bar chart illustrates the distribution of patient responses on the 7-point PGIC scale, where 1 indicates “very much worse” and 7 indicates “very much improved”.

On the PGIC scale, patients reported a median score of 5.5 (IQR 4–6), indicating an improvement ranging from moderate to substantial following treatment (Figure 1).

Additionally, all directions of shoulder range of motion demonstrated significant improvement following completion of the treatment program (Table 2).

**Figure 2:**
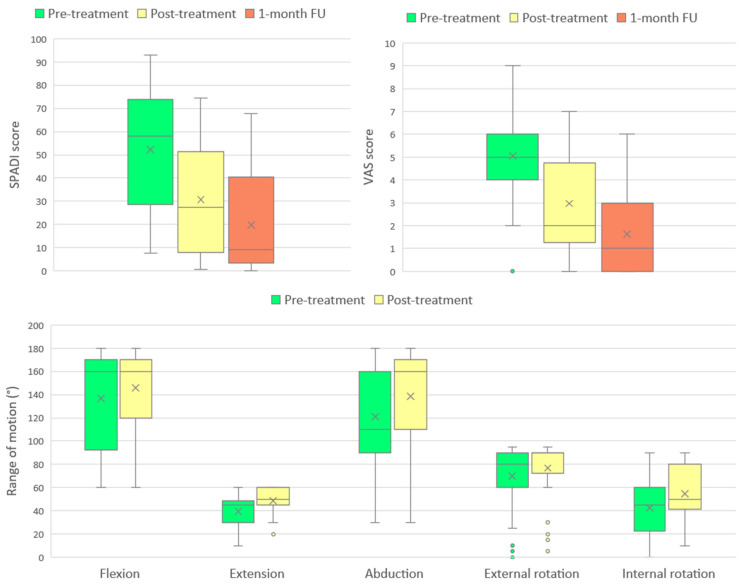
Changes in SPADI score, VAS score, and shoulder range of motion (ROM) across measured timepoints. Boxplots represent median, interquartile range, and outliers, with means indicated by “×”.

The progression of all monitored parameters, assessed at baseline, immediately after the treatment program, and at the one-month follow-up, is presented graphically in Figure 2.

Table 3 summarizes effect sizes and established MCID thresholds for each variable. All monitored outcomes exceeded the MCID values reported in current literature, indicating clinically meaningful changes.

A significant correlation was found between BMI and treatment-related improvement in selected domains; however, no significant correlation was observed between BMI and baseline values for any of these parameters (Table 4). Spearman’s rank correlation confirmed a significant relationship between higher BMI and greater improvement in internal rotation, SPADI, and extension.

**Figure 3:**
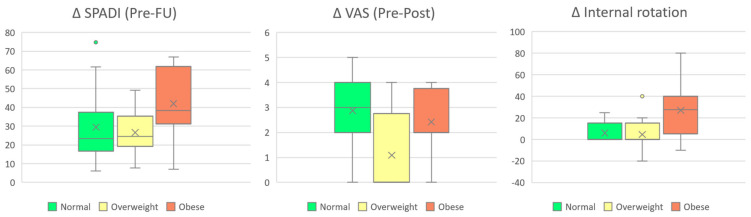
Changes in SPADI (Post–Follow-up), VAS (Pre–Post), and internal rotation stratified by BMI group. Boxes represent interquartile ranges (IQR), horizontal lines denote medians, and “×” symbols indicate group means.

In addition, the Kruskal–Wallis test revealed statistically significant differences between BMI groups for changes in internal rotation, SPADI, and VAS. While improvements in SPADI and internal rotation appeared to be more pronounced with increasing BMI, the relationship between BMI and changes in VAS scores from pre- to post-treatment was less consistent (Figure 3).

Linear regression models using BMI category as a categorical predictor revealed a significant effect on ΔVAS (Pre–Post), with overweight patients showing less improvement compared to normal-weight individuals (*p* = 0.008, R^2^ = 0.228). A trend toward statistical significance was also observed for ΔSPADI (Pre–Post), ΔAbduction, and ΔInternal Rotation (*p* = 0.078, 0.085, and 0.083, respectively), with BMI category explaining approximately 12–13% of the variance in these outcomes. Notably, ΔExtension showed a statistically significant association with BMI category (*p* = 0.035, R^2^ = 0.125), indicating greater improvement in obese patients.

The overall findings suggest that BMI may influence the therapeutic response to RSWT, particularly with respect to pain reduction and functional mobility.

The potential influence of comorbidities and affected-side dominance on treatment outcomes was also explored. Selected comorbidity domains showed statistically significant or clinically relevant associations with treatment response (Table 5). Several comorbidities were associated with poorer improvements in pain (e.g., digestive, cardiovascular), function (e.g., SPADI in musculoskeletal same-limb cases), or range of motion (e.g., flexion in endocrine and metabolic disorders). Interestingly, the presence of neurologic comorbidities was associated with greater improvement in internal rotation.

To further explore the clinical profile of patients, it was investigated whether the presence of selected comorbidities was associated with differences in BMI. Patients with neurologic (*p* = 0.029), metabolic (*p* = 0.037), and cardiovascular conditions (*p* = 0.044) were found to have significantly higher BMI values compared to those without these comorbidities.

## 4. Discussion

The present study demonstrated statistically significant improvements across all evaluated outcome measures following the RSWT treatment protocol. These findings are consistent with previous studies reporting that RSWT exerts beneficial effects on pain, functional capacity, disability, and shoulder mobility. However, direct quantitative comparisons between studies remain challenging, given the heterogeneity in study design, patient populations, and treatment protocols. Nevertheless, the magnitude of clinical improvement observed in this cohort falls within the range reported in the existing literature on RSWT efficacy. Additionally, most observed changes exceeded MCID thresholds and demonstrated large effect sizes, further supporting the practical relevance of the outcomes.

Importantly, the strong association between baseline pain levels and both functional limitations and clinical recovery trajectories is consistent with findings from other rehabilitation domains, including post-surgical and respiratory programs, where validated scoring systems have demonstrated similar patterns of alignment between pain reduction, improvement in range of motion, and global recovery status [54]. This reinforces the role of pain not only as a key symptom but also as a predictive marker of functional restoration, further justifying the use of integrated instruments such as SPADI and VAS in assessing treatment outcomes in adhesive capsulitis.

For instance, Kim et al. reported smaller post-treatment gains in VAS scores and shoulder abduction compared to those observed in our cohort. Conversely, they noted greater improvement in shoulder flexion, likely attributable to more limited baseline mobility [53]. Similarly, other studies involved participants with severely limited baseline flexion (88–106°) and elevated pain levels, leading to proportionally greater post-treatment improvements [20,22,23]. Nevertheless, despite undergoing therapy, participants in these studies did not reach the baseline flexion value of 160° observed in the present cohort.

Seyam et al. reported superior results in SPADI, flexion, abduction, and external rotation. This discrepancy may be explained by differences in patient characteristics, including a younger average age (45.3 ± 8.7 years), a small sample size (n = 15), and the inclusion of exclusively diabetic individuals [18,55].

Although both obesity and diabetes are recognized as risk factors for adhesive capsulitis, their specific influence on RSWT outcomes remains insufficiently investigated [32]. Based on available evidence, one might expect obesity to negatively impact treatment effectiveness [33,34]. Interestingly, our findings challenge this assumption, as patients with obesity demonstrated greater improvements in specific outcome measures compared to those with normal BMI.

This observation was further explored using regression models with BMI category as a predictor. While this supplementary analysis did not identify consistent effects across all outcomes, it supported a potential trend: patients with obesity appeared to achieve greater improvements in objective range of motion parameters, whereas overweight individuals tended to report less favorable subjective outcomes in pain perception.

One possible explanation for this discrepancy is that patients with higher BMI may have exhibited more limited baseline mobility, which could have amplified relative Pre–Post changes in range of motion. In contrast, the observed reduction in pain relief among overweight individuals may reflect heightened mechanical loading of periarticular structures, subclinical inflammation, or altered nociceptive processing associated with excess adiposity. These factors could contribute to persistent discomfort despite improvements in joint function, particularly in individuals with intermediate metabolic burden [56].

Although correlation analyses did not reveal a significant association between BMI and baseline ROM, the greater relative improvements observed among obese patients suggest that baseline restriction alone does not account for the observed effect. Interestingly, while several comorbidities—particularly endocrine, metabolic, and musculoskeletal conditions involving the treated limb—were linked to poorer ROM or SPADI outcomes, higher BMI exhibited an opposite pattern in selected ROM domains. Moreover, patients with neurologic comorbidities experienced the greatest improvements in internal rotation, indicating that not all comorbidities are detrimental to rehabilitation outcomes.

These findings suggest that the relationship between BMI and treatment response may involve distinct soft tissue characteristics—such as increased stiffness, altered hemodynamics, or localized fibrosis—rather than being solely driven by systemic disease burden. In neurologic patients, tissue rigidity resulting from disuse or spasticity may enhance responsiveness to the mechanical decompression and perfusion effects of radial shockwave therapy (RSWT) [57].

This observation may represent a form of the so-called “obesity paradox”, where individuals with elevated BMI show unexpectedly favorable responses to certain therapies. In this context, increased adiposity might influence local tissue architecture or metabolic reactivity in a way that enhances the therapeutic effects of RSWT, particularly in neurologically affected limbs [58].

Furthermore, the findings may reflect a distinct clinical phenotype of frozen shoulder associated with systemic dysmetabolism. Increasing evidence links the condition to low-grade inflammation and insulin resistance. From this perspective, patients with obesity could exhibit enhanced healing responses to therapies targeting fibrotic and hypovascular tissues—not purely due to biomechanical effects, but potentially also due to metabolically influenced repair mechanisms. This hypothesis remains speculative and would require direct investigation of inflammatory markers and body composition [59].

For subjective pain outcomes, digestive and cardiovascular comorbidities were associated with less improvement, aligning with the trend observed among overweight individuals in the regression model. This consistency supports the notion that systemic or metabolic burden—potentially including elevated BMI—may play a role in modulating pain perception and the analgesic response to RSWT.

From a biophysical perspective, increased subcutaneous fat implies a longer acoustic path from the skin to the target tissue, which could lead to increased energy dissipation and reduced transmission efficacy [60]. Nonetheless, the observed positive correlation between BMI and treatment response suggests that additional factors may be involved.

An alternative explanation for the observed correlation may lie in the protocol used to adjust RSWT intensity, which was tailored to each patient’s pain tolerance. This individualized approach could have allowed patients with higher BMI—who may present with reduced mechanical sensitivity—to tolerate greater pressure levels. The device permitted intensity settings of up to 6 bar, and it is plausible that obese individuals were able to reach higher therapeutic thresholds more easily. Given the known pain sensitivity associated with adhesive capsulitis, reaching optimal intensity levels is essential for effective treatment.

Although a definitive clinical relationship between pressure settings and tissue penetration depth has yet to be established, it is reasonable to hypothesize such a link. Existing studies suggest that higher RSWT pressures are associated with greater energy delivery and improved clinical outcomes [61,62]. In obese patients, deeper tissue penetration may be necessary to reach the fibrotic capsule effectively, and increased pressure could help overcome the damping effect of subcutaneous fat. When combined with better pain tolerance, this may result in more efficient energy transmission and thus improved therapeutic responses. These hypotheses warrant further investigation, ideally through studies directly measuring RSWT intensity, penetration depth, and clinical outcomes across BMI categories.

The principal limitation of this study is the lack of a control group, which precludes differentiation between treatment-induced and spontaneous improvements. In addition, ROM was not reassessed at follow-up, limiting conclusions regarding the durability of functional gains. Nonetheless, available evidence supports RSWT efficacy in adhesive capsulitis, and spontaneous resolution typically occurs over a prolonged, multi-year period. A reduction exceeding 40% in SPADI and VAS scores over a 10-day protocol is unlikely to reflect natural recovery alone [63]. Moreover, the clinical relevance of these improvements was supported by effect size calculations and comparison to published MCID thresholds.

The potential role of RSWT intensity as a mediator of treatment response—particularly in relation to BMI—underscores the importance of documenting and statistically adjusting for treatment parameters in future studies. Although tailoring therapy to individual tolerance is standard practice, accounting for anatomical variability (e.g., subcutaneous fat, muscle thickness) could enhance the interpretability of treatment effects. 

While the sample size of 40 was sufficient to detect significant within-subject changes, subgroup analyses by BMI would benefit from larger, more balanced cohorts. Inclusion of underweight individuals may also provide a more comprehensive perspective on BMI-related treatment effects. Future studies should also consider integrating comorbidity burden and affected limb dominance as covariates, even if their independent effect appears limited, to better control for potential confounding.

Future studies could incorporate musculoskeletal ultrasound to quantify local subcutaneous fat and evaluate its influence on energy delivery. Such objective measurements may help personalize RSWT protocols and improve treatment outcomes in obese patients—a group frequently affected by adhesive capsulitis and often less responsive to conventional or surgical interventions [33,34].

Despite its limitations, the present study offers valuable preliminary insights into the potential interaction between BMI and RSWT effectiveness in adhesive capsulitis. These findings may inform the design of future trials aimed at optimizing treatment strategies for distinct patient subgroups.

## 5. Conclusions

The present study suggests the effectiveness of RSWT in alleviating pain, disability, and range of motion limitations in patients diagnosed with adhesive capsulitis. Improvements across all evaluated outcomes were not only statistically significant but also clinically meaningful, as demonstrated by large effect sizes and consistent exceeding of MCID thresholds. The observed positive association between BMI and selected treatment outcomes suggests that RSWT may represent a particularly effective and well-tolerated non-invasive therapeutic option for individuals with obesity—a subgroup frequently affected by adhesive capsulitis and often showing reduced responsiveness to both conventional conservative interventions and surgical procedures. Importantly, BMI was not associated with worse baseline clinical status, supporting the interpretation that greater improvements reflect a true treatment effect rather than regression to the mean. While most comorbidities were linked to poorer clinical outcomes, notably in domains of pain and function, neurologic comorbidities were associated with greater improvements in internal rotation. This raises the possibility that enhanced responsiveness in obese patients may be partially modulated by neurologic factors or by mechanical and tissue-related characteristics more prevalent in this subgroup. Further studies are warranted to explore how body composition, neuromuscular status, and systemic health interact to shape the therapeutic response to RSWT.

Further research is warranted to elucidate the underlying physiological mechanisms and to confirm the true extent of BMI’s impact on RSWT responsiveness, with a view toward refining treatment protocols and optimizing patient-specific rehabilitation strategies. While limited by its retrospective design and sample size, this study generates new hypotheses regarding BMI-related responsiveness to RSWT, which should be tested in future controlled trials.

## Figures and Tables

**Table 1:** Summary of treatment outcomes at baseline, immediately post-treatment, and at one-month follow-up. Data are presented as median and interquartile range (IQR). The Wilcoxon signed-rank test was used for pairwise comparisons between baseline and post-treatment values. For outcomes assessed at three time points (baseline, post-treatment, and follow-up), the Friedman test was applied. Statistically significant differences (*p* < 0.05) are shown in bold.

	SPADI	VAS
Pre-treatment	57.95 (29.03–73.80)	5 (4–6)
Post-treatment	27.30 (8.30–49.45)	2 (1.75–4.25)
∆ Pre–Post (%)	−42.78 ± 41.88	−45.99 ± 33
1-month FU	9.25 (3.63–38.05)	1 (0–3)
∆ Pre–FU (%)	−67.32 ± 27.08	−70.91 ± 34.51
Wilcoxon (Pre–Post)	***p* < 0.001**	***p* < 0.001**
Wilcoxon (Pre–FU)	***p* < 0.001**	***p* < 0.001**
Friedman	χ^2^ = 66.23 ***p* < 0.001**	χ^2^ = 64.79 ***p* < 0.001**

Δ Pre–Post (%) and Δ Pre–FU (%) represent percentage changes from baseline to post-treatment and to follow-up, respectively. For each patient, values were calculated as (Timepoint − Pre-treatment)/Pre-treatment × 100, and the table reports the average change ± standard deviation across all patients. Negative values indicate improvement for outcomes where lower scores reflect better status.

**Table 2:** Summary of ROM measurements performed at baseline and immediately after completion of treatment program. Values are presented as median and interquartile range (IQR). The Wilcoxon signed-rank test was used to compare pre- and post-treatment values. Statistically significant differences (*p* < 0.05) are indicated in bold.

	Flexion	Extension	Abduction	External Rotation	Internal Rotation
Pre-treatment (°)	160 (97.5–170)	45 (30–46.25)	110 (90–160)	80 (60–90)	45 (27.5–60)
Post-treatment (°)	160 (120–170)	50 (45–60)	160 (110–170)	90 (77.5–90)	50 (43.75–80)
∆ Pre–Post (%)	9.14 ± 24.24	38.47 ± 82.85	23.01 ± 58.11	26.66 ± 63.57	66.03 ± 159.99
Wilcoxon	***p* < 0.001**	***p* < 0.001**	***p* < 0.001**	***p* < 0.001**	***p* < 0.001**

Δ Pre–Post (%): calculated for each patient as (Post-treatment − Pre-treatment)/Pre-treatment × 100. The values reported in the table represent the average percentage change ± standard deviation across all patients.

**Table 3:** Effect sizes and clinical relevance of treatment outcomes. Cohen’s d values represent the magnitude of change between pre- and post-treatment measurements. Clinical relevance was assumed when the observed change exceeded published thresholds for MCID.

Parameter	Cohen’s d	Effect Size Interpretation	Approximate MCID	Change Exceeded MCID
Extension	1.86	Large	10–15°	Yes
VAS	1.82	Large	1.5–2.0 points	Yes
Flexion	1.77	Large	15–20°	Yes
External rotation	1.27	Large	10–15°	Yes
SPADI	1.26	Large	8–13 points	Yes
Abduction	1.13	Large	15–20°	Yes
Internal rotation	0.93	Medium–Large	10–20°	Yes

Note. MCID values were based on the published literature for shoulder conditions and represent the minimum change considered clinically meaningful by patients.

**Table 4:** Relationship between BMI and improvement in clinical outcome measures following RSWT. Change scores were calculated as differences between baseline and either post-treatment or 1-month follow-up. Spearman’s rank correlation was used to assess the association between BMI and continuous change scores. The Kruskal–Wallis test evaluated differences in treatment response across three BMI categories (normal weight, overweight, and obese). Statistically significant values (*p* < 0.05) are presented in bold.

	Spearman	Kruskal-Wallis
∆ Internal rotation	ρ = 0.390 ***p* = 0.014**	H = 9.14 ***p* = 0.010**
∆ SPADI (Pre–FU)	R_s_ = −0.339 ***p* = 0.032**	H = 6.06 ***p* = 0.048**
∆ Extension	ρ = 0.327 ***p* = 0.039**	H = 4.39 *p* = 0.111
∆ VAS (Pre–Post)	ρ = −0.107 *p* = 0.511	H = 8.75 ***p* = 0.013**
∆ VAS (Pre–FU)	ρ = −0.037 *p* = 0.821	H = 2.41 *p* = 0.299
∆ Abduction	ρ = 0.285 *p* = 0.075	H = 1.80 *p* = 0.407
∆ SPADI (Pre–Post)	ρ = 0.222 *p* = 0.169	H = 4.8 *p* = 0.124
∆ External rotation	ρ = 0.160 *p* = 0.313	H = 1.52 *p* = 0.467
∆ Flexion	ρ = 0.115 *p* = 0.479	H = 0.72 *p* = 0.697

**Table 5:** Summary of comorbidity-related effects on treatment outcomes. Only variables with *p* < 0.1 are shown. Statistically significant values (*p* < 0.05) are presented in bold.

Factor	Outcome Variable	*p*-Value	Effect
Neurology	∆ Internal rotation	**0.003**	More improvement
Musculoskeletal (same limb)	∆ SPADI (Pre–FU)	**0.049**	Less improvement
Digestive	∆ VAS (Pre–Post)	0.0621	Less improvement
Endocrinology	∆ Flexion	0.076	Less improvement
Metabolic	∆ Flexion	0.092	Less improvement
Cardiovascular	∆ VAS (Pre–FU)	0.095	Less improvement

Note. “Less improvement” refers to a lower median change in the outcome measure among patients with the comorbidity compared to those without, whereas “more improvement” refers to a higher median change in the outcome among patients with the comorbidity. “Musculoskeletal (same limb)” indicates a musculoskeletal condition affecting the same upper limb as the treated shoulder.

## Data Availability

The data presented in this study are available on request from the corresponding author. The data are not publicly available due to privacy and ethical restrictions.

## Abbreviations

The following abbreviations are used in this manuscript:

RSWT	Radial shockwave therapy
BMI	Body mass index
SPADI	Shoulder pain and disability index
VAS	Visual analog scale
ROM	Range of motion
PGIC	Patient global impression of change
IQR	Interquartile range

## References

[1] Tang C.-W., Lin T.-Y., Shen P.-C., Tang F.-T. (2024). Evaluating the Effectiveness of Ultrasound-Guided Subacromial-Subdeltoid Bursa and Coracohumeral Ligament Corticosteroid Injections With and Without Physiotherapy in Adhesive Capsulitis Treatment. Biomedicines.

[2] Le H.V., Lee S.J., Nazarian A., Rodriguez E.K. (2016). Adhesive capsulitis of the shoulder: Review of pathophysiology and current clinical treatments. Shoulder Elb..

[3] Sheridan M.A., Hannafin J.A. (2006). Upper Extremity: Emphasis on Frozen Shoulder. Orthop. Clin. N. Am..

[4] Pérez-Mármol J.M., García-Ríos M.C., Ortega-Valdivieso M.A., Cano-Deltell E.E., Peralta-Ramírez M.I., Ickmans K., Aguilar-Ferrándiz M.E. (2017). Effectiveness of a fine motor skills rehabilitation program on upper limb disability, manual dexterity, pinch strength, range of fingers motion, performance in activities of daily living, functional independency, and general self-efficacy in hand osteoarthritis: A randomized clinical trial. J. Hand Ther..

[5] Sporea C., Morcov M.V., Morcov M., Mirea A. (2023). Effectiveness of Passive Movement Training in Patients with Cerebral Palsy: A Comparative Analysis of Robot-Assisted Therapy and Electrical Stimulation in Hand Rehabilitation. Balneo PRM Res. J..

[6] Haaland K.Y., Mutha P.K., Rinehart J.K., Daniels M., Cushnyr B., Adair J.C. (2012). Relationship Between Arm Usage and Instrumental Activities of Daily Living After Unilateral Stroke. Arch. Phys. Med. Rehabil..

[7] Chan H., Pua P., How C. (2017). Physical therapy in the management of frozen shoulder. Singap. Med. J..

[8] Tache-Codreanu D.-L., David I., Tache-Codreanu A.-M., Sporea C., Burcea C.-C., Blendea D.C., Morcov M.-V., Cioca I.E. (2025). rESWT in Shoulder Periarthritis: Does the Protocol Intensity Matter?—A Quasi-Experimental Non-Randomized Comparative Study. Life.

[9] Neviaser A.S., Neviaser R.J. (2011). Adhesive Capsulitis of the Shoulder. J. Am. Acad. Orthop. Surg..

[10] Tache-Codreanu D.-L., Trăistaru M.R. (2024). The Effectiveness of High Intensity Laser in Improving Motor Deficits in Patients with Lumbar Disc Herniation. Life.

[11] Uzun Ö., Özcan D.S., Arslan H.B., Bilir E.E., Şentürk B., Tezen Ö. (2025). Impact of TECAR therapy on pain and function in adhesive capsulitis: A randomized controlled trial. Lasers Med. Sci..

[12] Raeisi M., Mohammadi H.K., Heshmatipour M., Tarrahi M.J., Taheri N. (2022). Effect of Transfer Energy Capacitive and Resistive Therapy on Shoulder Pain, Disability, and Range of Motion in Patients with Adhesive Capsulitis: A Study Protocol for a Randomized Controlled Trial. J. Chiropr. Med..

[13] Burcea C.-C., Oancea M.-D., Tache-Codreanu D.-L., Georgescu L., Neagoe I.-C., Sporea C. (2024). The Benefits of a Rehabilitation Program Following Medial Patellofemoral Ligament Reconstruction. Life.

[14] Hasanloei M.A.V., Gholizadeh S., Shafiei-Irannejad V. (2024). 25th National and 11th International Annual Congress on Research and Technology of Iranian Medical Sciences Students, Urmia, Iran, 5–7 September, 2024. Iran. Biomed. J..

[15] 1Burcea C.-C., Bobu V., Ferechide D., Neagoe I.C., Lupușoru G.E., Sporea C., Lupușoru M.O.D. (2023). New methodological aspects in rehabilitation after proximal humerus fracture. Balneo PRM Res. J..

[16] Stefanova D., Angelcheva M., Bogdanova S. (2022). A Comparative Study on the Effect of Extracorporeal Shock Wave Therapy and Interferential Electrotherapy in Periarthritis Humeroscapularis. Proceedings of the International Scientific Congress “Applied Sports Sciences” 2022.

[17] Madzharova R., Simeonov E. (2020). Comparing the Effect of Conventional Physiotherapy and Radial Shockwave Therapy in Patients with Capsulitis Adhesive on Shoulder Joint. EurasianUnionScientists.

[18] Seyam M.K., Moubarak E.E., Rahim Shaik A. (2018). The Effect of Extracorporeal Shock Wave Therapy for Patients with Diabetic Frozen Shoulder. Majmaah J. Health Sci..

[19] Yehia R.M., ElMeligie M.M. (2022). Effectiveness of extracorporeal shockwave therapy for frozen shoulder in perimenopausal diabetic women. Biomed. Hum. Kinet..

[20] Naggar T.E.D.M.E., Maaty A.I.E., Mohamed A.E. (2020). Effectiveness of radial extracorporeal shock-wave therapy versus ultrasound-guided low-dose intra-articular steroid injection in improving shoulder pain, function, and range of motion in diabetic patients with shoulder adhesive capsulitis. J. Shoulder Elb. Surg..

[21] Elerian A.E., Rodriguez-Sanz D., Elsherif A.A., Dorgham H.A., Al-Hamaky D.M.A., El Fakharany M.S., Ewidea M. (2021). Effectiveness of Shock Wave Therapy versus Intra-Articular Corticosteroid Injection in Diabetic Frozen Shoulder Patients’ Management: Randomized Controlled Trial. Appl. Sci..

[22] Lee S., Lee S., Jeong M., Oh H., Lee K. (2017). The effects of extracorporeal shock wave therapy on pain and range of motion in patients with adhesive capsulitis. J. Phys. Ther. Sci..

[23] Salem M.M.I., Abdelaal A.A.M., El-Fiky A.A.-R., Ebid A.A., Battecha K.H., Thabet A.A.E.-M., Mousa G. (2024). High-Intensity Laser Therapy Versus Shock Wave Therapy in the Management of Diabetic Frozen Shoulder. Pharmacophore.

[24] Manske R.C., Prohaska D. (2008). Diagnosis and management of adhesive capsulitis. Curr. Rev. Musculoskelet. Med..

[25] Navarro-Ledesma S. (2025). Frozen Shoulder as a Metabolic and Immune Disorder: Potential Roles of Leptin Resistance, JAK-STAT Dysregulation, and Fibrosis. J. Clin. Med..

[26] Pérez-Montilla J.J., Guzmán-García R., Pruimboom L., Navarro-Ledesma S. (2025). Does Leptin and Insulin Levels Influence Pain and Disability in Subjects with Frozen Shoulder? A Cross-Sectional Study. Eur. J. Pain.

[27] Navarro-Ledesma S., Hamed-Hamed D., Pruimboom L. (2024). A new perspective of frozen shoulder pathology; the interplay between the brain and the immune system. Front. Physiol..

[28] Vettor R., Milan G., Rossato M., Federspil G. (2005). Review article: Adipocytokines and insulin resistance. Aliment. Pharmacol. Ther..

[29] Shah A., Mehta N., Reilly M.P. (2008). Adipose Inflammation, Insulin Resistance, and Cardiovascular Disease. J. Parenter. Enter. Nutr..

[30] Austin D.C., Gans I., Park M.J., Carey J.L., Kelly J.D. (2014). The association of metabolic syndrome markers with adhesive capsulitis. J. Shoulder Elb. Surg..

[31] Pietrzak M. (2016). Adhesive capsulitis: An age related symptom of metabolic syndrome and chronic low-grade inflammation?. Med. Hypotheses.

[32] Kim J.-H., Baek J.-Y., Han K.-D., Kim B.-S., Kwon H.-S. (2024). Higher body mass index increases the risk of shoulder adhesive capsulitis in young adults: A nationwide cohort study. J. Shoulder Elb. Surg..

[33] Barbosa F., Swamy G., Salem H., Creswell T., Espag M., Tambe A., Clark D. (2019). Chronic adhesive capsulitis (Frozen shoulder): Comparative outcomes of treatment in patients with diabetes and obesity. J. Clin. Orthop. Trauma.

[34] Alhussain A., Alhathlol H.A., Alsharif A.A., Alsikhan K.M., Almagushi N.A. (2025). Assessing the influence of obesity on rotator cuff repair surgical and functional outcomes: A meta-analysis. JSES Int..

[35] Tache-Codreanu D.-L., Bobocea L., David I., Burcea C.-C., Sporea C. (2024). The Role of the Six-Minute Walk Test in the Functional Evaluation of the Efficacy of Rehabilitation Programs After COVID-19. Life.

[36] Liang S., Wu J., Chen W., Shih C. (2008). An in-vitro experiment on lysing adipose tissue by shock waves. J. Med. Biol. Eng..

[37] Lin H.-H., Huang T.-F., Ma H.-L., Liu C.-L. (2013). Body mass index and active range of motion exercise treatment after intra-articular injection in adhesive capsulitis. J. Chin. Med. Assoc..

[38] Flegal K.M., Kit B.K., Orpana H., Graubard B.I. (2013). Association of All-Cause Mortality with Overweight and Obesity Using Standard Body Mass Index Categories: A systematic review and meta-analysis. JAMA.

[39] Tache-Codreanu D.-L., David I., Blendea D.C., Tache-Codreanu A.-M., Morcov M.-V., Sporea C. (2025). Impact of a Multidisciplinary Functional Recovery Program on Post-Lung Transplant Outcomes: A One-Year Follow-Up. Balneo PRM Res. J..

[40] Ioppolo F., Tattoli M., Di Sante L., Attanasi C., Venditto T., Servidio M., Cacchio A., Santilli V. (2012). Extracorporeal Shock-Wave Therapy for Supraspinatus Calcifying Tendinitis: A Randomized Clinical Trial Comparing Two Different Energy Levels. Phys. Ther..

[41] McAteer J.A., Evan A.P. (2008). The Acute and Long-Term Adverse Effects of Shock Wave Lithotripsy. Semin. Nephrol..

[42] Testa G., Vescio A., Perez S., Consoli A., Costarella L., Sessa G., Pavone V. (2020). Extracorporeal Shockwave Therapy Treatment in Upper Limb Diseases: A Systematic Review. J. Clin. Med..

[43] Huskisson E.C. (1974). Measurement of Pain. Lancet.

[44] Roach K.E., Budiman-Mak E., Songsiridej N., Lertratanakul Y. (1991). Development of a shoulder pain and disability index. Arthritis Care Res..

[45] Hurst H., Bolton J. (2004). Assessing the clinical significance of change scores recorded on subjective outcome measures. J. Manip. Physiol. Ther..

[46] Patients’ Global Impression of Change (PGIC) Scale. https://chiro.org/LINKS/OUTCOME/Patients_Global_Impression_of_Change.pdf.

[47] Tache-Codreanu D.-L., Tache-Codreanu A. (2024). Acting and Dancing during the COVID-19 Pandemic as Art Therapy for the Rehabilitation of Children with Behavioural Disorders Living in Socially Disadvantaged Environments. Children.

[48] Young J., Bucher H.C. (2004). A conventional proof of efficacy requires an intent-to-treat analysis. J. Clin. Epidemiol..

[49] Kelly A.-M. (2001). The minimum clinically significant difference in visual analogue scale pain score does not differ with severity of pain. Emerg. Med. J..

[50] Roy J., MacDermid J.C., Woodhouse L.J. (2009). Measuring shoulder function: A systematic review of four questionnaires. Arthritis Care Res..

[51] Michener L.A., McClure P.W., Sennett B.J. (2002). American Shoulder and Elbow Surgeons Standardized Shoulder Assessment Form, patient self-report section: Reliability, validity, and responsiveness. J. Shoulder Elb. Surg..

[52] Charan J., Biswas T. (2013). How to Calculate Sample Size for Different Study Designs in Medical Research?. Indian J. Psychol. Med..

[53] Kim J., Oh C., Yoo J., Yim J. (2022). Applying Focused and Radial Shock Wave for Calcific Tendinitis of the Shoulder: Randomized Controlled Study. Phys. Ther. Rehabil. Sci..

[54] Tache-Codreanu D.-L., David I., Popp C.G., Bobocea L., Trăistaru M.R. (2024). Successfully physical therapy program for functional respiratory rehabilitation after lung transplant surgery—Case report. Rom. J. Morphol. Embryol..

[55] Mohammad S., Bhattacharjee J., Tzaneva V., Hutchinson K.A., Shaikh M., da Silva D.F., Burger D., Adamo K.B. (2023). The Influence of Exercise-Associated Small Extracellular Vesicles on Trophoblasts In Vitro. Biomedicines.

[56] Eichwald T., Talbot S. (2020). Neuro-Immunity Controls Obesity-Induced Pain. Front. Hum. Neurosci..

[57] Hones K.M., Hao K.A., Cueto R.J., Wright J.O., King J.J., Wright T.W., Friedman R.J., Schoch B.S. (2023). The Obesity Paradox: A Nonlinear Relationship Between 30-Day Postoperative Complications and Body Mass Index After Total Shoulder Arthroplasty. J. Am. Acad. Orthop. Surg..

[58] Corrado B., Di Luise C., Iammarrone C.S. (2019). Management of Muscle Spasticity in Children with Cerebral Palsy by Means of Extracorporeal Shockwave Therapy: A Systematic Review of the Literature. Dev. Neurorehabilit..

[59] Poursaeed F., Tahan N., Manshadi F.D., Bagheban A.R.A. (2021). Effects of Extracorporeal Shockwave Therapy on Clinical and Neurophysiological Indices of Spasticity Inpatients with Upper Motor Neuron Lesions: A Systematic Review and Meta-analysis. J. Rehabil..

[60] Nagla S., Abou-Farha M., El-Abd A., Gameel T., Eltatawy H. (2022). Efficacy of extracorporeal shockwave lithotripsy, with modified position of the machine head in the treatment of lower calyceal stones in obese patients. Urol. Ann..

[61] Bai X., Li Z., Zhang H., Wang C., Yu J., Fu Y., Liao W., Yu Y., Qu W., Li J. (2014). Study on the Accurate Effects of Radial Shock Wave Therapy Equipment. Zhongguo Yi Liao Qi Xie Za Zhi.

[62] Arıcan M., Turhan Y., Karaduman Z.O. (2019). Dose-related Effect of Radial Extracorporeal Shockwave Therapy (rESWT) on Lateral Epicondylitis in Active Patients: A Retrospective Comparative Study. Iran. Red Crescent Med. J..

[63] Vastamäki H., Kettunen J., Vastamäki M. (2011). The Natural History of Idiopathic Frozen Shoulder: A 2- to 27-year Followup Study. Clin. Orthop. Relat. Res..

